# Adaptive expression responses in the Pol-γ null strain of *S. pombe *depleted of mitochondrial genome

**DOI:** 10.1186/1471-2164-8-323

**Published:** 2007-09-15

**Authors:** Zhaoqing Chu, Juntao Li, Majid Eshaghi, R Krishna Murthy Karuturi, Kui Lin, Jianhua Liu

**Affiliations:** 1 Systems Biology, Genome Institute of Singapore, Singapore 138672, Singapore; 2 Computational and Mathematical Biology, Genome Institute of Singapore, Singapore 138672, Singapore; 3 College of Life Sciences, Beijing Normal University, Beijing 100875, China; 4 Department of Biochemistry, Yong Loo Ling School of Medicine, National University of Singapore, Singapore 117597, Singapore

## Abstract

**Background:**

DNA polymerase γ(Pol-γ) has been shown to be essential for maintenance of the mitochondrial genome (mtDNA) in the petite-positive budding yeast *Saccharomyces cerevisiae*. Budding yeast cells lacking mitochondria exhibit a slow-growing or petite-colony phenotype. Petite strains fail to grow on non-fermentable carbon sources. However, it is not clear whether the Pol-γ is required for mtDNA maintenance in the petite-negative fission yeast *Schizosaccharomyces pombe*.

**Results:**

We show that disruption of the nuclear gene *pog1*^+ ^that encodes Pol-γ is sufficient to deplete mtDNA in *S. pombe*. Cells bearing *pog1Δ *allele require substantial growth periods to form petite colonies. Mitotracker assays indicate that *pog1Δ *cells are defective in mitochondrial function and EM analyses suggest that *pog1Δ *cells lack normal mitochondrial structures. Depletion of mtDNA in *pog1Δ *cells is evident from quantitative real-time PCR assays. Genome-wide expression profiles of *pog1Δ *and other mtDNA-less cells reveal that many genes involved in response to stimulus, energy derivation by oxidation of organic compounds, cellular carbohydrate metabolism, and energy reserve metabolism are induced. Conversely, many genes encoding proteins involved in amino acid metabolism and oxidative phosphorylation are repressed.

**Conclusion:**

By showing that Pol-γ is essential for mtDNA maintenance and disruption of *pog1*^+ ^alters the genome-wide expression profiles, we demonstrated that cells lacking mtDNA exhibit adaptive nuclear gene expression responses in the petite-negative *S. pombe*.

## Background

Mitochondria are essential organelles that generate ATP through the respiratory chain to provide energy for many biochemical reactions and cellular processes in eukaryotic cells. Mitochondria are one of the organelles that contain its own genetic material, the mitochondrial genome (mtDNA). It is thought that mitochondria evolved over millions of years after originating from bacteria that had invaded eukaryotic cells. It is thus not surprising that Pol-γ has a lineage similar to prokaryotic DNA polymerase A in *E. coli *[1,2]. A nuclear-DNA encoded gene of the catalytic subunit of Pol γ (*POLG1 *in human and *MIP1 *in *Saccharomyces cerevisiae*) has been shown to be essential for mtDNA stability [3,4].

The mitochondrial genomes in many mammalian systems are small when compared to their corresponding nuclear genomes. Human mtDNA, for example, is only ~16.6 kb in length and rodent mtDNA is ~16.3 kb [5-9]. Mutations in either human mtDNA or *POLG1 *have been implicated in a number of diseases such as Leigh's syndrome, Alpers syndrome, and ophthalmoplegia [10]. In *S. cerevisiae*, the size of mtDNA is relatively large, ~85.8 kb in size. Cells depleted of mtDNA (ρ°) have been isolated through the treatment with ethidium bromide which results in the formation of tiny or petite colonies in *S. cerevisiae *[11]. The petite mutants fail to grow on media containing non-fermentable carbon sources owing to a lack of mitochondrial functions. Petites are not readily isolated from wild type *Schizosaccharomyces pombe *using ethidium bromide treatment [12]. However, some unidentified nuclear mutations have facilitated the isolation of cells depleted of mtDNA [13]. The isolated petites exhibited severely retarded growth phenotypes that impeded further genetic analyses of these petite strains.

In this study, we show that the nuclear gene *pog1*^+ ^(we designate the Pol-γ-encoding gene as *pog1*^+^, because the *mip1*^+ ^designation used in *S. cerevisiae *has already been used for a gene encoding a WD repeat protein in *S. pombe*) is essential for mtDNA maintenance. Significantly, cells bearing a *pog1Δ *allele are capable of forming petite colonies after extended incubation times under optimal growth conditions. The *pog1Δ *cells display multiple defects such as non-uptake of mitotracker dyes, impaired mitochondrial structures, and depletion of mtDNA. Genome-wide expression profiling of mtDNA-less cells reveals that many genes encoding proteins involved in response to stimulus, carbohydrate metabolism, and energy reserve metabolism are induced. On the other hand, many genes encoding proteins involved in amino acid metabolism and oxidative phosphorylation are repressed. The result indicates an adaptive expression response in petite-negative mtDNA-less *S. pombe *strains.

## Results

### DNA polymerase γ is important to support normal growth in *S. pombe*

**Figure 1:**
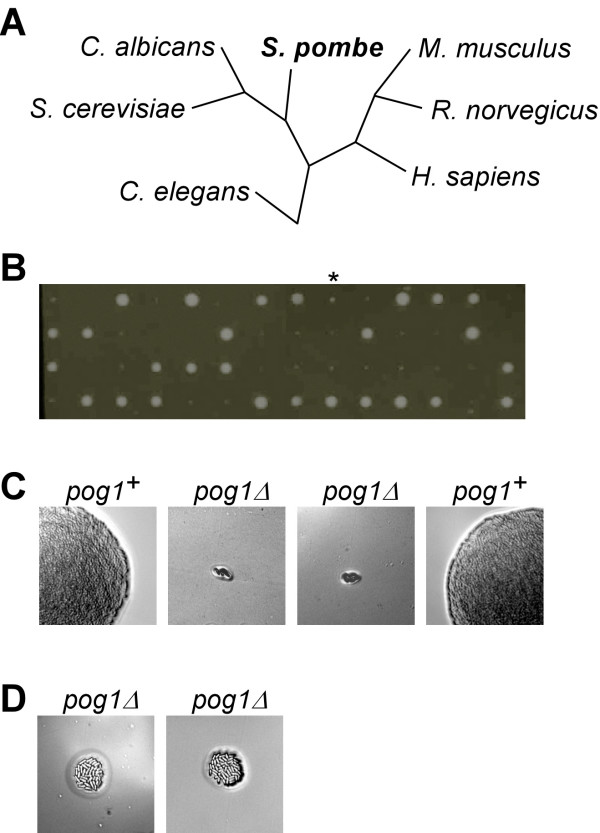
Disruption of the gene encoding Pol-γ. (A) The phylogenic tree of Pol-γ. (B) Tetrad dissection analysis of spores generated from *pog1*^+^*/pog1Δ *diploid cells after incubation at 30°C for ~5 days. The asterisk indicates that an uppermost wild type spore in the 9^th ^tetrad was implanted into the agar and thus grows slower than other wild type spores. (C) The magnified view of a tetrad in (B). Genotypes of individual spores are indicated. (D) *pog1Δ *spores form petite colonies after a lengthy incubation term (~15 days) at 30°C.

We extracted protein sequences of the DNA polymerase γ (SPCC24B10.22, designated as *pog1*^+ ^for Pol-γ) using the *S. pombe *genome in the public database [14]. BLAST analysis of the *S. pombe *Pol-γ revealed its homolog in various other species such as *Homo sapiens*, *Rattus norvegicus*, *Mus musculus*, *Caenorhabditis elegans*, *Candida albicans*, and *S. cerevisiae*. Phylogenic analysis based on protein sequences showed that *S. pombe *Pol-γ, like these in *S. cerevisiae *or *C. albicans*, is very similar to those in mammalian systems (Figure 1A).

To investigate the function of the Pol-γ, we performed gene deletion analysis using a PCR-mediated approach [15]. Diploid transformants that formed colonies on plates without uracil supplement were isolated for further validation of gene disruption. The genotype of diploid *pog1*^+^/*pog1Δ *strains was validated by PCR-assays using sequence-specific primers (Data not shown). To further examine whether *pog1*^+ ^is essential for viability, we performed tetrad dissection analysis. Of ~15 asci/tetrads dissected on YES plates, all tetrads displayed a 2:2 segregation of normal growing spores versus non-growing spores after growth at 30°C for ~5 days, except for 1 tetrad in which one of the wild type spores was implanted deep into the agar and thus impeded its normal growth (figure 1B and 1C). This result indicates that *pog1*^+ ^is essential for vegetative growth. Consistent with this, spores that formed normal-sized colonies were unable to grow on uracil-dropout plates (Data not shown). PCR assays confirmed that those spores possessed a wild type allele of *pog1*^+^, but not a deletion allele (Data not shown). However, after extended incubation terms, we found the *pog1Δ *spores were able to form petite colonies (Figure 1D). This result indicates that Pol-γ is not essential for viability, but is important for normal growth rate in *S. pombe*.

### Cells bearing a *pog1Δ *allele exhibit multiple growth phenotypes

**Figure 2:**
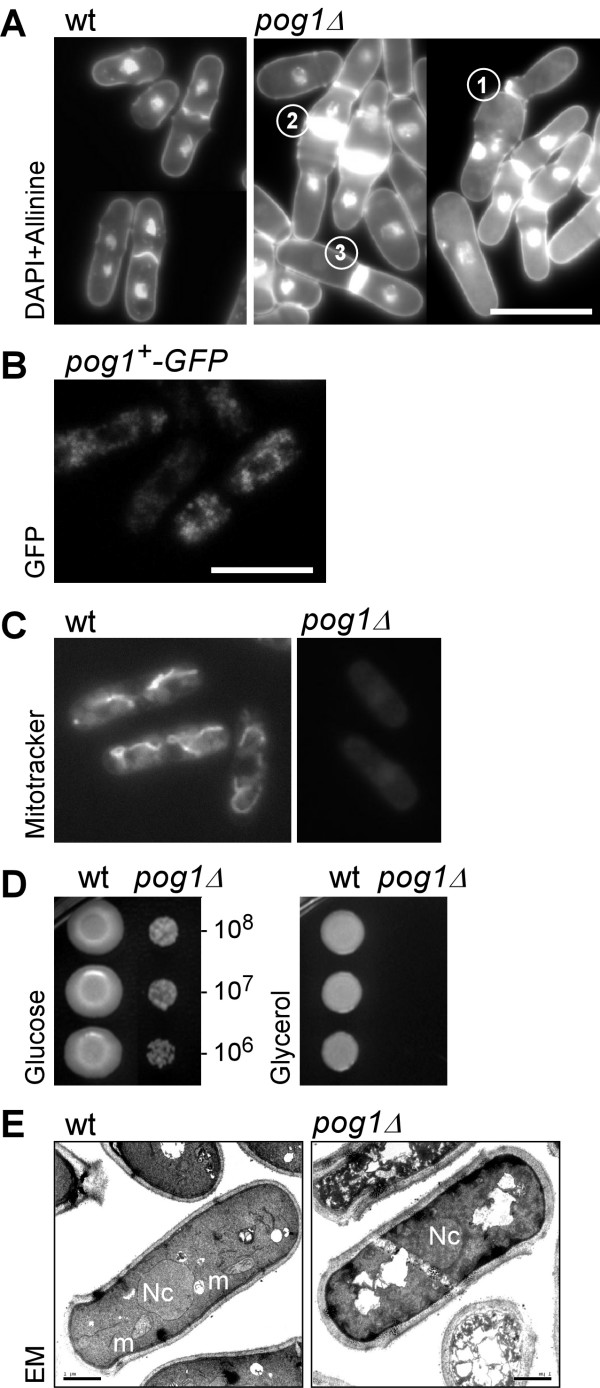
Growth phenotypes of *pog1Δ *cells. (A) Abnormal cellular morphologies of *pog1Δ *cells. Circled number 1 and 2 indicate dumb-bell cell shapes; number 2 and 3 indicate cells with excessive division septum deposition; number 3 indicates anucleate cells. (B) Subcellular localization of Pog1-γ. Cells bearing a sole copy of *pog1*^+^*-GFP *were examined for GFP localization using fluorescence microscopy. (C) Live cells stained with mitochondria-specific compound MitoTracker. (D) Approximately 5 μl of 10-fold series diluted cells were spotted onto standard EMM medium (glucose) and glycerol medium (glycerol) and incubated at 30°C for 5–7 days. (E) EM images of wild type and *pog1Δ *cells. Nc and m stand for nucleus and mitochondrion, respectively.

To investigate phenotypes of the *pog1Δ *strain, the mutant cells were subjected to DAPI and aniline staining to visualize DNA and cell wall, respectively. Wild type cells were cylindrical shaped ~4–5 μm in diameter and ~12–14 μm in length (Figure 2A). The *pog1Δ *strain were typically dumbbell shaped (Figure 2A, see number 1 and 2), had abnormally thick septa (Figure 2A, see number 2 and 3), multiple septa (Figure 2A, see number 2) and anucleate cells (Figure 2A, see number 3). The multiple phenotypes of the *pog1Δ *strain are consistent with the notion that mitochondria are "power plants" that generate energy to support many biochemical reactions and cellular processes.

We next investigated the Pol-γ subcellular localization in wild type cells. For this reason, we constructed a strain bearing a sole copy of *pog1*^+^-GFP that exhibited no abnormal growth phenotypes, suggesting that C-terminus tagging of Pol-γ does not affect its biological function. The GFP-tagged Pol-γ showed the subcellular localization resembling to that of mitochondria previously reported [16,17] (Figure 2B).

Functional mitochondria are capable of mitotracker uptake, a mitochondrion-selective fluorescent dye [18]. To test whether mitochondria were defective in *pog1Δ *cells, we stained the cells with mitotracker fluorescent dyes. As shown in Figure 2C, mitotracker fluorescent dyes aggregated to outline the functional mitochondrial structures in wild type cells. On the other hand, *pog1Δ *cells failed to show any fluorescent dye uptake, indicating that mitochondria are not functional in *pog1Δ *cells (Figure 2C). Cells with mitochondrial defects would fail to grow in media containing only non-fermentable carbon sources such as ethanol or glycerol [13]. To examine the growth ability of the *pog1Δ *strain on non-fermentable carbon sources, serial dilution of wild type and *pog1Δ *cultures were inoculated on plates containing either fermentable carbon source (*i.e*., glucose) or non-fermentable carbon source (*i.e*., glycerol). Both wild type and *pog1Δ *cells were capable of growing on glucose medium, although *pog1Δ *cells grew substantially slower than wild type cells (Figure 2D). On the other hand, *pog1Δ *cells failed to grow on plates containing glycerol, consistent with the conclusion that mitochondria are not functional in *pog1Δ *cells (Figure 2D).

To investigate whether the mitochondrial malfunction in *pog1Δ *cells was associated with disrupted mitochondrial structures, we thus performed electron microscopic (EM) analysis. EM images clearly showed the nuclear structure in wild type and *pog1Δ *strains (Figure 2E). However, normal mitochondrial structures were only apparent in wild type cells, but not *pog1Δ*, suggesting that the mitochondrial architecture had been disrupted (Figure 2E). These results indicate that disruption of Pol-γ is sufficient to abolish normal mitochondrial structure and consequently mitochondrial function.

### Mitochondrial DNA is depleted in *pog1Δ *cells

To investigate the role of Pol-γ in maintaining the copy number of mtDNA, we performed real-time quantitative PCR (qPCR) using the Syber Green assay on whole DNA extracted from various strains. Due to different efficiencies of primer pairs in qPCR assay, the identical concentration of template DNA could give rise to different Ct-values (threshold cycle) from different primer pairs. For adequate estimation of the copy number of the samples, multiple pairs of primers specific to either mtDNA or nuclear genome were utilized in the Syber Green assays. The median of the Ct values from ~40 primer pairs specific to mtDNA was used to approximate the copy number of mtDNA. On the other hand, the median Ct value of ~60 primer pairs specific to nuclear DNA was used to estimate the copy number of the nuclear DNA. The higher the copy number of the sample, the sooner accumulated product would be detected in the real-time qPCR assay as a significant increase in fluorescence, and the lower the Ct value.

**Figure 3:**
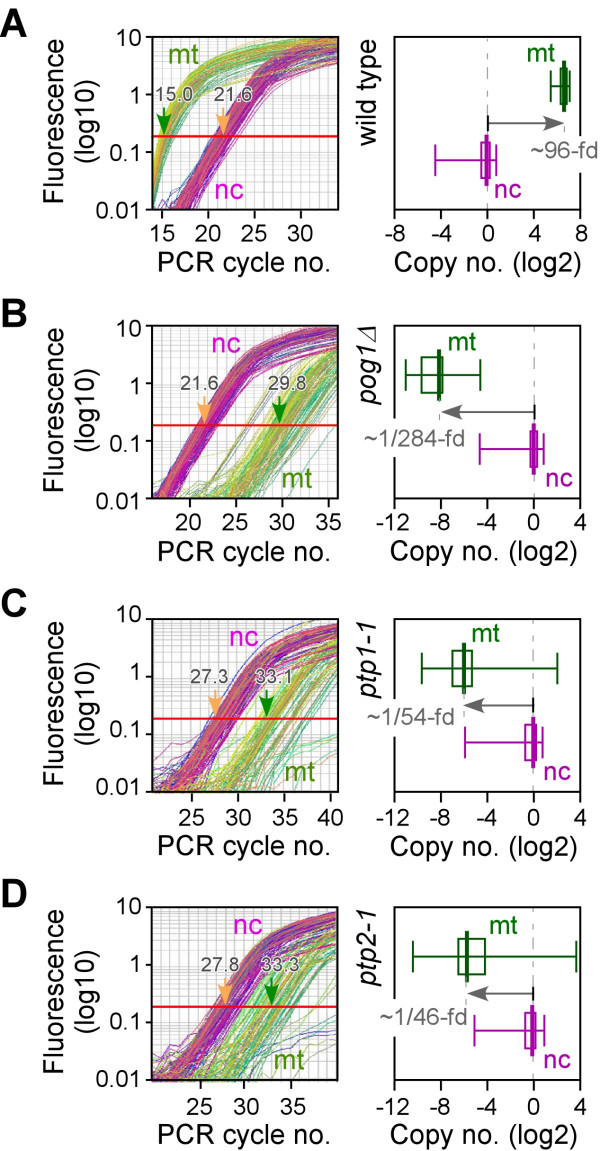
Comparisons of the copy number of mtDNA and nuclear DNA in various *S. pombe *strains. The left panel is the plot of fluorescence versus PCR cycle number. The arrows indicate the median of Ct-values for either mtDNA (mt; in green) or nuclear DNA (nc; in purple). The right panel is the box-plot representing the distribution Ct-values by primer pairs either specific to mtDNA (in green) or nuclear DNA (in purple): the minimal (left-end bar), the first quantile (left side of the box), median (central think bar), the third quantile (right side of the box), and the maximal (right-end bar). The copy number of nuclear DNA in various strains is set to 1 as reference for the copy number of mtDNA. The assays were carried out using total DNA samples extracted from wild type (A), *pog1Δ *(B), *ptp1-1 *(C), and *ptp2-1 *(D) cells as indicated.

In wild type cells, the median Ct value for the mtDNA and nuclear DNA was 15.0 and 21.6, respectively, using the Syber Green assay (Figure 3A, left panel). That is, the median Ct value of mtDNA was 6.6-cycles less than that of the nuclear DNA. For easy comparison, 6.6-cycles difference could be converted into ~96.6 (equivalent to 2^6.6^)-fold difference. In other words, the copy number of mtDNA was ~96.6-fold higher than that of nuclear DNA in wild type cells (Figure 3A, right panel). This result indicates that there are ~100 copies of mtDNA in a haploid G1-phase cell (a G1-cell would contain 1 copy of nuclear DNA). Given that the cell-cycle length was mostly occupied by G2-phase (a haploid G2-cell would contain two copies of nuclear DNA) under optimal growth conditions in *S. pombe *[19], an active growing *S. pombe *cell would thus contain ~100–200 copies.

We next investigated the copy number of mtDNA in *pog1Δ *cells. A majority of *pog1Δ *cells were found to possess 2C-DNA contents, as seen in wild type cells (Data not shown), suggesting no changes in nuclear DNA profiles. According to the real-time qPCR assay, the median Ct value of mtDNA and nuclear DNA was found to be 29.8 and 21.6, respectively, in *pog1Δ *cells (Figure 3B). That is to say, the median Ct value of mtDNA was 8.2-cycles more than that of the nuclear DNA, opposite to those in wild type cells (Figure 3A). Therefore, the copy number of mtDNA was ~1/284 (or 2^-8.2^)-fold less than that of the nuclear DNA in *pog1Δ *cells (Figure 3B). Approximately 150–300 *pog1Δ *cells would share one copy of mtDNA. This result indicates that mtDNA was dramatically depleted in *pog1Δ *cells.

It has been reported that no mtDNA could be detected by Southern blot analysis in *ptp2-1 *and *ptp1-1 *cells [13]. We thus applied both strains as negative control for the Syber Green assay. As shown in Figure 3C and 3D, the median Ct value of mtDNA was much higher than that of nuclear DNA in either *ptp2-1 *or *ptp1-1 *strains, indicating that the Syber Green assay is adequate for determination of mtDNA and nuclear DNA copy numbers. Based on the assay, it was estimated that ~25–50 *ptp2-1 *or *ptp1-1 *cells would share one copy of mtDNA, suggesting that mtDNA was depleted in *ptp *mutant cells.

### Differentially expressed genes in the *pog1Δ *strain

Whole-genome expression profiling of mutant strains is a useful approach to assess genetic phenotypes of various strains [20]. To investigate differential expressions in mtDNA-less cells, we performed expression profile analysis in *pog1Δ*, *ptp1-1*, and *ptp2-1 *cells using the *S. pombe *genome-wide DNA microarray [19]. To this end, total RNA extracted from various strains was reverse transcribed in presence of cyanine dye Cy5-coupled dUTP. Cy5-labeled cDNA was subjected to microarray hybridized against a Cy3-labeled common reference cDNA (wild type cells). For reproducibility at least 3 independent assays were performed.

**Figure 4:**
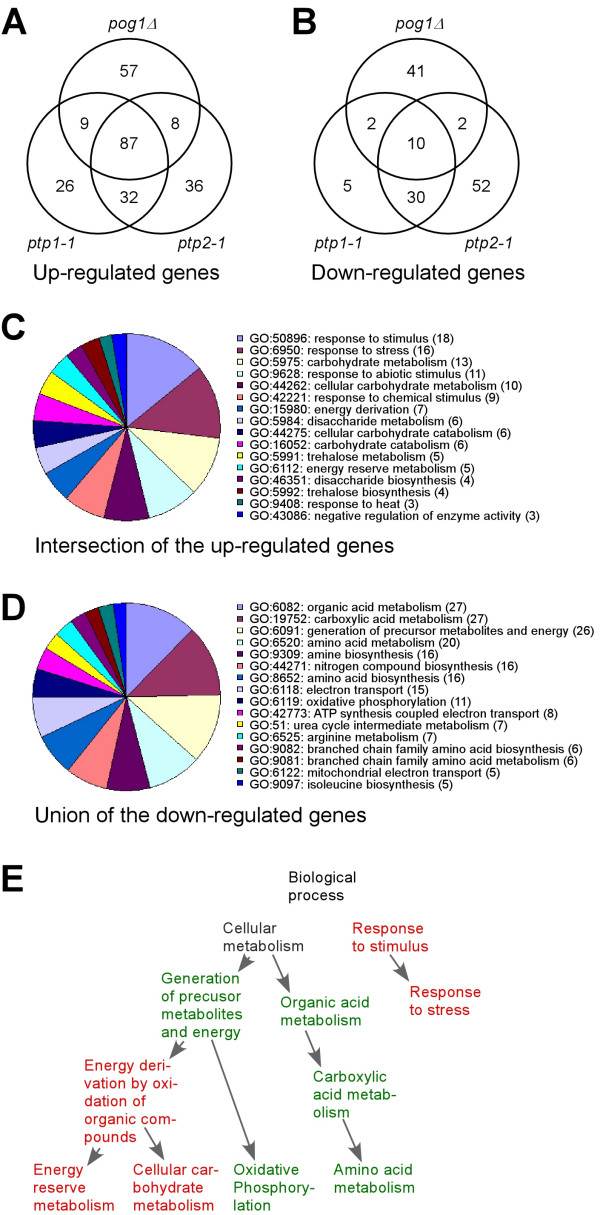
Characteristics of differentially expressed genes in mtDNA-less strains. (A) and (B) Venn diagrams of the up-regulated genes (3-fold or greater) and down-regulated genes (1/3-fold or less), respectively, in *pog1Δ*, *ptp1-1*, and *ptp2-1 *cells compared to the wild type cells. (C) Enriched gene-ontology categories in the list of the intersection of the up-regulated genes in the mtDNA-less strains. A gene-ontology category is considered if three genes or more in the tested gene list are involved in the category. (D) Enriched gene-ontology categories in the list of the union of the down-regulated genes in the mtDNA-less strains. (E) GO Graph. The relationship of some enriched GO categories is shown. Categories in red and green indicate those enriched with up- and down-regulated genes, respectively.

Expression profiles of mtDNA-less strains were compared with the wild type self-self hybridizations for identification of differentially expressed genes using SAM analysis [21]. As a result, approximately 161, 154, and 163 genes whose expression levels showed to be up-regulated for 3-fold or more in *pog1Δ*, *ptp1-1*, and *ptp2-1*, respectively (Figure 4A). Approximately 87 up-regulated genes [for the complete list of genes, see Additional File 1] were common in *pog1Δ*, *ptp1-1*, and *ptp2-1 *strain, suggesting a universal pattern of expression responses in the mtDNA-less cells. The 87 up-regulated genes showed to significantly enrich for genes involved in response to various stimuli and stress factors (GO:6950; GO:9408; GO:9628; GO:42221; and GO:50896), carbohydrate metabolism (GO:5975; GO:5984; GO:5991; GO:6112; and GO:44262), catabolism and biosynthesis (GO:5992; GO:16052; GO:44275; and GO:46351), and others (GO:15980 and GO:43086) (p-value < 0.001) (Figure 4C).

Approximately 55, 47, and 94 genes were found to be down-regulated for at least 3-fold or more in *pog1Δ*, *ptp1-1*, and *ptp2-1*, respectively, compared to those in wild type cells (Figure 4B). Although only ~10 genes were common to all three strains, the union of 142 genes appeared to be significantly enriched in the down-regulated genes of individual mtDNA-less strains when the threshold was reduced to 2-fold differences (Data not shown). These 142 down-regulated genes [for the complete list of genes, see Additional File 1] were found to enrich those involved in amino acids and protein metabolism and biosynthesis (GO:51; GO:6082; GO:6091; GO:6520; GO:6525; GO:8652; GO:9081; GO:9082; GO:9097; GO:9309; GO:19752; and GO:44271) and oxidative phosphorylation and respiratory chain reactions (GO:6118; GO:6119; GO:6122; and GO:42773) (p-value < 8.5× 10^-7^) (Figure 4D).

Comparison of expression profiles of cells depleted of mtDNA in *S. cerevisiae *[22,23] and human Namalwa cell lines [24] yielded a common theme that genes encoding enzymes involved in glycolytic pathways and stress response pathways are induced in ρ° cells, suggesting an evolutionarily conserved transcriptional response mechanism to the depletion of mitochondrial function (Figure 4E).

### Repression of genes involved in energy generation in *pog1Δ *cells

**Figure 5:**
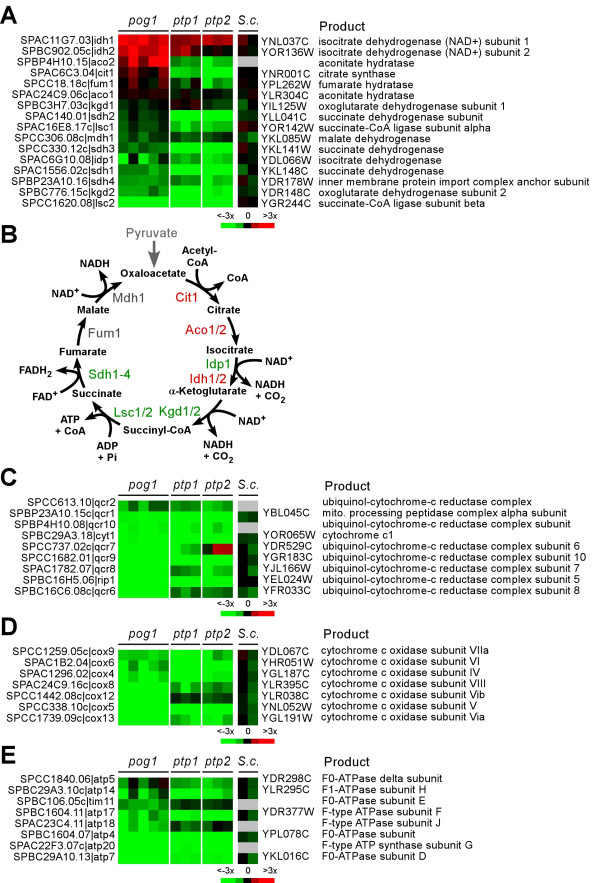
Expression profiles of *pog1Δ*, *ptp1-1*, and *ptp2-1 *cells. (A) Expression profiles of genes encoding TCA cycle enzymes. The phasogram of gene expression profiles (mtDNA-less cells versus wild type cells) is shown in which rows indicate genes and columns indicate individual repeats (for *S.c*., columns represent different cell densities). *S. pombe *gene names are listed in the left and *S. cerevisiae *(*S.c*.) are listed in the right. Induced expression levels are shown in red and repressed are in green (*S.c*. data are based on the study by Epstein et al.). No changes are in black and no data are in grey. The color key is shown at the bottom. (B) TCA cycle. Genes induced are indicated in red while genes repressed are in green. (C-E) Expression profiles of genes encoding proteins involved in cytochrome c reductase, oxidase, and ATP synthase complexes, respectively. The profiles are displayed as in (A).

Enzymes involved in TCA cycle and cellular respiration are required to function in mitochondria. Many of these enzymes are encoded by nuclear DNA. Only a few subunits of the cytochrome c oxidase (COX) and ATPase complexes are encoded by the mtDNA in *S. pombe*, for example, *cox1, cox2, cox3, atp6, atp8*, and *atp9 *[25]. To investigate whether nuclear genes encoding respiratory proteins were expressed in *pog1Δ*, *ptp1-1 *and *ptp2-1 *cells, we extracted expression profiles of these genes from the genome-wide profiles. For this reason, steady-state levels of 16 transcripts encoding enzymes involved in the TCA cycle were first extracted and compared with those of ρ° cells in *S. cerevisiae *based on the study by Epstein et al. [23] (Figure 5A). The degree of expression level alterations in *S pombe *mtDNA-less *pog1Δ, ptp1-1*, and *ptp2-1 *strains was more dramatic than those found in ρ° cells of *S. cerevisiae *(Figure 5A). Significantly, expression levels of enzymes in part of the TCA cycle such as isocitrate dehydrogenase subunits Idh1p and Idh2p were elevated in all *S. pombe *mtDNA-less strains. The levels of citrate synthase Cit1p, aconitate hydratase Aco1p and Aco2p were elevated only in *pog1Δ *cells, but reduced in *ptp1-1 *or *ptp2-1 *cells. Conversely, levels of succinate-CoA ligase subunits Lsc1p and Lsc2p, succinate dehydrogenase subunits Sdh1-4p, and isocitrate dehydrogenase were reduced in all three *S. pombe *mtDNA-less strains (Figure 5A and 5B). The result indicates that not all genes encoding enzymes involved in TCA cycle are down-regulated in the mtDNA-less strain.

We subsequently examined expression profiles of genes encoding cytochrome c reductase and oxidase complexes. As shown in Figure 5C and 5D, expression levels of transcripts function in both reductase and oxidase were repressed in *pog1Δ*, *ptp1-1*, and *ptp2-1 *cells, except for the levels of *cqr7*^+ ^in *ptp2-1*, consistent with a lack of mitochondrial function. In addition, expression levels of ATP synthase complexes were also repressed (Figure 5E). These results suggested that genes encoding proteins involved in energy generation in mitochondria were largely repressed in cells depleted of mtDNA.

## Discussion

The fission yeast *S. pombe *is known as the petite-negative that can not tolerate the lost of mtDNA, in contrast to the budding yeast *S. cerevisiae *[12]. However, some nuclear mutations are known to allow growth of mtDNA-less *S. pombe *cells [13]. In this study, we show that an evolutionarily conserved Pol-γ is essential for maintenance of mtDNA in *S. pombe*. Spores bearing a *pog1Δ *allele form petite colonies after extended incubation terms (Figure 1D and 1E). We have further shown that *pog1Δ *cells exhibit multiple growth phenotypes (Figure 2) and are depleted of mtDNA, similar to the *ptp *mutants (Figure 3). Thus, the petite-negative *S. pombe *mtDNA-less cells do not cease to grow on medium containing fermentable carbon courses, although the growth is substantially slow and exhibit severe growth phenotypes.

We show that *pog1Δ *cells depleted of mtDNA possess no normal mitochondrial structures when compared with cells containing mtDNA in EM analyses (Figure 2E). This result suggests that mtDNA is required for maintaining normal mitochondrial structures in *S. pombe*. Mitochondrial respiratory complexes including cytochrome c oxidase and ATPase are encoded by both nuclear and mitochondrial genomes [26,27]. In *S. pombe*, mtDNA encodes at least 3 subunits of the cytochrome c oxidase (COX) complex and 3 subunits of the ATPase complex [25]. Our result suggests that without these COX and ATPase subunits cells fail to assemble membranes with mitochondrial potential. Consistent with this, *pog1Δ *cells fail to uptake mitochondria-specific dye or mitotracker due to a lack of mitochondrial membrane potential (Figure 2C).

We show that, on average, each haploid cell contains ~100–200 copies of mtDNA based on the real-time qPCR analysis (Figure 3A). This is consistent with the observation of number of mitochondria-specific GFP foci reported by Matsuyama et al. [17] and in this study (Figure 2B). In *pog1Δ *cells, the mtDNA drops to ~1/300–1/150 copies per cell. Similarly, the mtDNA in *ptp1-1 *or *ptp2-1 *cells also drops to ~1/50–1/25 copies per cell (Figure 3C and 3D). The result supports the notion that these strains are depleted of mtDNA. Residues detected by mtDNA-specific primers in the real-time qPCR assay are probably represented the non-specific products; or other nuclear DNA polymerases in *S. pombe *could inefficiently rescue the lack of Pol-γ by replicating mtDNA in *pog1Δ *cells. Alternatively, *pog1Δ *spores may carry over the mtDNA from the diploid mother cells during sproulation. The mtDNA copy number is substantially diluted during the growth of *pog1Δ *cells. However, it is not clear why the *ptp *mutant strains display higher mtDNA background than the *pog1Δ *(Figure 3B–D).

Genome-wide expression profiling of mtDNA-less cells revealed that the nuclear genes encoding carbohydrate metabolism and response to stimulus are induced by at least 3-fold. This result suggests that cells with impaired mitochondrial function attempt to maintain their growth competence potential (Figure 4E). In addition, cells lacking mtDNA appear to undergo extensive oxidative metabolism, consistent with the notion that respiratory deficiency induces peroxisome biogenesis [22,23]. On the other hand, genes encoding proteins involved in amino acids metabolism and energy production are down-regulated. This result suggested that mtDNA-less cells are adapted for slow growth and repress genes that are involved in mitochondrial function. Indeed, the majority of the nuclear genes encoding enzymes involved in TCA cycle and almost all genes involved in cytochrome c reductase and oxidase complexes are repressed (Figure 5), similar to those seen in ρ° cells in *S. cerevisiae*.

## Conclusion

We have demonstrated that Pol-γ is essential for mtDNA maintenance. Cells bearing a *pog1Δ *allele are viable but grow substantially slower in the petite-negative *S. pombe*. Expression profiling of *pog1Δ *and other mtDNA-less mutant cells indicates that genes involved in glycolytic pathways and stress response are induced, common to budding yeast and human cell line systems [22-24]. Taken together, we propose that a common theme of adaptive expression responses to the disruption of mtDNA occur in both petite-positive (*eg., S. cerevisiae*) and petite-negative (*eg., S. pombe*) cells.

## Methods

### Strains and media

*S. pombe *strains YJL1000 (*h*^-^*/h*^+^*ade6-M210/ade6-M216 leu1-32/leu1-32 ura4-D18/ura4-D18*), YJL1001 (*h*^-^*/h*^+^*pog1*^+^*/pog1Δ ::ura4*^+^*ade6-M210/ade6-M216 leu1-32/leu1-32 ura4-D18/ura4-D18*), YJL1002 (*h*^-^*pog1Δ ::ura4*^+^*ade6*^-^*leu1-32 ura4-D18*), YJL1003 (*h*^-^*pog1*^+^*-GFP::ura4*^+^*leu1-32 ura4-D18*), YJL1004 (*h*^-^*leu1-32 ura4-D18*), YJL1005 (*h- leu1-32*); PHP14 (*h*^-^*ade6-M216 leu1-32 ptp1-1*), and PHP4 (*h*^-^*ade6-M210 ptp2-1*) were used in this study. Standard fission yeast growth media YES and EMM were used [28]. To test growth ability on non-fermentable carbon source, glycerol medium (glycerol replaces glucose in EMM medium) was used. Cultures were grown at 28–30°C unless otherwise mentioned.

Diploid strain YJL1000 was subjected to transformation using PCR fragments generated using primer pairs (SPCC24B10.22_UPF, 5'-TACTTCTTTGAGTTGCTGGTCTTGGAATAGTGTCGCTAAAGTAAATTCCAGTTGGGCGTCCATTGCTCAAGTACTTCATGTAACCCTCACTAAAGGGAAC-3'; and SPCC24B10.22_DNR, 5'-CAATGTCATAACTATCCCTAAAATTATCCTAACCGAAAAACACATTTTTTAACTAAAATAAATTATTCTTTTGCTGATCACTCACTATAGGGCGAATTGG-3') on plasmid pJH1000 (pBlueScript containing a HindIII fragment harboring *ura4*^+^). YJL1001 colonies bearing heterozygous allele of *pog1Δ ::ura4*^+^*/pog1*^+ ^were selected on uracil-drop plates. Tetrads arising from sporulation of YJL1001 were dissected on a MSM system dissection microscope (Singer Instruments, UK) and one of the *ura4*^+ ^spores collected as labeled YJL1002. Primers (SPCC24B10.22_A, 5'-ACATTTAACTGTTTATACTTGC-3'; SPCC24B10.22_B, 5'-AGAGCAGGTGATAAGTATTGAA-3'; SPCC24B10.22_C, 5'-TCTAACAAAGTCCCCATACC-3'; SPCC24B10.22_D, 5'-GATCCGTTCTGTTTCTATATGT-3') were used in PCR assays for validation of *pog1Δ ::ura4*^+ ^alleles. PCR fragments that were synthesized by primer pairs (SPCC24B10.22_TAF, 5'-ATGTTAAAGCTACTACTTCAGCAGAGATTACAGAAGAAGACAAAAAAAACATTGCATATTTAAAAGCACAAGCATTTTACTGGATCCCCGGGTTAATTAA-3'; and SPCC24B10.22_DNR) on template pJH (pBlueScript containing a GFP coding sequence in pJH1000) were transformed into strain YJL1004 to yield YJL1003 containing a sole copy of *pog1*^+^*-GFP*.

### Microscopic and FACS analyses

#### Fluorescence microscopy

Cells were fixed by adding one tenth volume of concentrated formaldehyde (Sigma-Aldrich) for 10 min and washed with PBS. DNA and cells walls were stained with 4'-6-Diamidino-2-phenylindole (DAPI) and Aniline blue (Sigma-Aldrich) respectively, for fluorescence microscopic analysis (Leica, Wetzlar, Germany), respectively.

#### Electron microscopy

Briefly, cells were fixed in 3% glutaraldehyde contained in 0.1 M Na cacodylate buffer (pH 7.4) containing 5 mM CaCl_2_, 5 mM MgCl_2_, and 2.5% sucrose for 1 hr at 25°C with gentle agitation; spheroplasted; embedded in 2% ultra low temperature agarose (prepared in water); cooled; and subsequently cut into small pieces (<1 mm^3^) [29]. The cells were then post-fixed in 1% OsO_4_/1% potassium ferrocyanide contained in 0.1 M cacodylate/5 mM CaCl_2 _buffer (pH 7.4) for 30 min at room temperature. The blocks were washed thoroughly four times with ddH_2_O, 10 min total; transferred to 1% thiocarbohydrazide at room temperature for 3 min; washed in ddH_2_O (four times, 1 min each); and transferred to 1% OsO_4_/1% (pH 7.4) for an additional 3 min at room temp. The cells were then washed four times with ddH_2_O (15 min total), en bloc stained in Kellenberger's uranyl acetate for 2 h to overnight, dehydrated through a graded series of ethanol, and subsequently embedded in Spurr resin. Ultrathin sections were cut on a Jung Reichert ultramicrotome and examined with a transmission electron microscope (JEM1010, JEOL) at 100 kV.

#### Tetrad dissection microscopy

Approximately 15 tetrads were dissected with a tetrad dissection MSM System microscope (Singer Instrument Inc., UK) on YES plates, incubated at 30°C. Colonies were replica-plated onto uracil-dropout plates for determination of *pog1Δ ::ura4*^+ ^or *ura4-D18 *markers.

#### Fluorescence-activated cell sorting (FACS) analyses

To measure DNA content, cells were quickly spun out and resuspended in ice-cold 70% ethanol and digested with RNase A (Roche, Basel, Switzerland) in 50 mM sodium citrate overnight, and briefly washed with PBS. DNA was stained with propidium iodine (Sigma-Aldrich). Fluorescence intensities of individual cells were measured by flow cytometry using a BD FACScan (DB Biosciences, Franklin Lakes, NJ).

#### MitoTracker assay

To determine functional mitochondrion, vegetative growth cells were harvested by centrifugation at 2,000 rpm for 5 min at 4°C and washed with water. Subsequently, the cells were resuspended in solution containing 100 nM MitoTracker (Invitrogen Corporation, Carlsbad, CA) and incubated for 15 min at room temperature. Before examining under a fluorescence microscope (Leica, Wetzlar, Germany), the stained cells were washed with water for at least three times.

### Isolation of nucleic acids

#### DNA extraction

10-ml culture was centrifuged at 2500–3000 rpm for 2 min. The cell pellet was resuspended in 1-ml distilled water and transferred into a 2-ml eppendorf tube. The tube was then subjected to a brief centrifugation for 30 sec at 12 Krpm in a microcentrifuge. The cells were resuspended in 0.5-ml sorbitol buffer (1.2 M sorbitol/0.1 M EDTA) containing Zymolyase (US Biological, Swampscott, MA) at a final concentration of 2 mg/ml and incubated at 37°C for 30 min. The spheroplasts were collected by centrifugation at 14 Krpm for 5 min and resuspended in 0.5-ml TES buffer (50 mM Tris/500 mM EDTA/100 mM NaCl, pH 8.0). 50 μl of both 10% SDS and 20 mg/ml proteinase K (Roche, Mannheim, Germany) were added into the spheroplasts and incubated at 65°C for 30 min with gentle mixing in a thermomixer (Eppendorf AG, Hamburg, Germany) at 500–600 rpm. The tube was transferred onto ice. 200-ml 5 M KCl was later added into the tube, the contents mixed, and the sample incubated on ice for additional 30 min. Bulky proteins were removed by centrifugation at 14 Krpm for 30 min at 4°C. DNA was washed with 80% cold ethanol and air dried. Resuspended DNA was subjected to DNase-free RNase (Roche) treatment and then extraction with phenol and chloroform and ethanol-precipitation. Resuspended DNA was quantified using a NanoDrop UV photo-spectrometer (NanoDrop Technologies, Wilmington, DE).

#### RNA extraction

Total RNA was extracted using acid phenol and treated with RNase-free DNase (Ambion Inc., Austin, TX) for 20 min at room temperature. For RT-qPCR analysis, the RNA was subsequently used as template to synthesize cDNA using reverse transcriptase (Invitrogen Co., Carlsbad, CA) with poly-d(T)_20 _primers according to manufacturer's guide. cDNA was cleaned by extraction with phenol and precipitation using ethanol. Then RNA pellet was resuspended in DEPC-treated distilled water and quantified.

### Real-time qPCR analysis

Aliquots of ~20-ng total DNA were added to each well in a 96-well plate containing Syber Green master mix (Applied Biosystems, Foster City, CA). Triplicate PCR reactions for each gene-specific primer pairs [for a list of primer sequences, see Additional File 2] were carried out according to manufacturer's instruction using a quantitative real-time PCR machine (ABI PRISM^® ^Sequence Detection System, Applied Biosystems) with an analytic software SDS2.2 (Applied Biosystems). In brief, it was programmed for 1 cycle at 95°C for 10 min, followed by 40 cycles at 95°C for 10 min and 60°C for 1 min. An additional cycle of 15 sec at 95°C, 60°C, and 95°C each was used to monitor the dissociation dynamics of PCR products. Standardized cycle threshold (Ct) of each PCR reaction was collected if the dissociation curve indicated no multiple products.

### Microarray fabrication and hybridization

50-mer oligonucleotides (Proligo Biochemie GmbH, Hamburg, Germany) at a normalized concentration of 40 nM each in 96-well plates were mixed with equal volumes of 0.3× SSC buffer and transferred to 384-well plates using a BioMed FX liquid handling robot (Beckman Coulter Inc., Fullerton, CA). The oligonucleotides were subsequently spotted onto poly-Lysine coated glass slides at random positions using a spotting machine (GeneMachines, San Carlos, CA). Spotted slides were post-processed as described previously [19] before hybridization with fluorescent dye-labeled cDNA.

Approximately 20 μg of total RNA were used to synthesize cDNA in presence of fluorescent dye Cy3- or Cy5-dUTP using a reverse transcriptase kit (Invitrogen). Labeled cDNA was cleaned by washing on a microcon-YM30 column (Millipore, Billerica, MA). Equal amounts of Cy3- and Cy5-labeled cDNA was pooled and co-hybridized to a microarray slide using a EasyHyb buffer (Roche, Basel, Switzerland) in a hybridization chamber (GeneMachines) or a MAUI^® ^mixer (BioMicro Systems, Salt Lake City, UT) at 42°C overnight. The slide was subsequently washed in a series of buffers containing various concentration of SSC (2× – 0.2×) with or without 1% SDS and dried by centrifugation before scanning.

### Microarray data acquisition and processing

Microarray slides were scanned using a GenePix scanner (Axon Instruments, Union City, CA) at wavelengths 635 and 532 μm with a resolution of 10 μm controlled by GenePix Pro4 softwares (Axon Instruments). GPR (GenePix Result) files were generated and globally normalized based on a median of ratios by GenePix Pro. Ratios (i.e., median of ratios) and absolute intensity (i.e., median of absolute intensity) of each spot were collected only if the absolute intensity of spots (in either channel) was 2-fold or higher than the background to ensure good data quality.

#### Intensity normalization

Intensity normalization assumed that the average absolute intensity *Cy3 *of individual features in an array is a constant *C*, for a series of microarray hybridizations with a common reference (labeled with Cy3-dye). Each array is related to this constant *C *by a factor *k*, i.e.C=k[(∑i=1nCy3i)/n]
 MathType@MTEF@5@5@+=feaafiart1ev1aaatCvAUfKttLearuWrP9MDH5MBPbIqV92AaeXatLxBI9gBaebbnrfifHhDYfgasaacH8akY=wiFfYdH8Gipec8Eeeu0xXdbba9frFj0=OqFfea0dXdd9vqai=hGuQ8kuc9pgc9s8qqaq=dirpe0xb9q8qiLsFr0=vr0=vr0dc8meaabaqaciaacaGaaeqabaqabeGadaaakeaacqWGdbWqcqGH9aqpcqWGRbWAdaWadaqaamaabmaabaWaaabCaeaacqWGdbWqcqWG5bqEcqaIZaWmdaWgaaWcbaGaemyAaKgabeaaaeaacqWGPbqAcqGH9aqpcqaIXaqmaeaacqWGUbGBa0GaeyyeIuoaaOGaayjkaiaawMcaaiabc+caViabd6gaUbGaay5waiaaw2faaaaa@41E2@, where *C *is set to 2500 (arbitrary unit) for convenience, *i *is an individual feature on an array, and *n *is a total number of available features (*n *≤ 9858). To remove outliers during determining factor *k *for an array, selected features in factor *k *estimation were only those whose log-intensity (log_10_*Cy3*) are within the 2σ range, i.e.|log⁡10Cy3−μ^|<2σ^
 MathType@MTEF@5@5@+=feaafiart1ev1aaatCvAUfKttLearuWrP9MDH5MBPbIqV92AaeXatLxBI9gBaebbnrfifHhDYfgasaacH8akY=wiFfYdH8Gipec8Eeeu0xXdbba9frFj0=OqFfea0dXdd9vqai=hGuQ8kuc9pgc9s8qqaq=dirpe0xb9q8qiLsFr0=vr0=vr0dc8meaabaqaciaacaGaaeqabaqabeGadaaakeaacqGG8baFcyGGSbaBcqGGVbWBcqGGNbWzdaWgaaWcbaGaeGymaeJaeGimaadabeaakiabdoeadjabdMha5jabiodaZiabgkHiTGGaciqb=X7aTzaajaGaeiiFaWNaeyipaWJaeGOmaiJaf83WdmNbaKaaaaa@3FDA@, where μ^
 MathType@MTEF@5@5@+=feaafiart1ev1aaatCvAUfKttLearuWrP9MDH5MBPbIqV92AaeXatLxBI9gBaebbnrfifHhDYfgasaacH8akY=wiFfYdH8Gipec8Eeeu0xXdbba9frFj0=OqFfea0dXdd9vqai=hGuQ8kuc9pgc9s8qqaq=dirpe0xb9q8qiLsFr0=vr0=vr0dc8meaabaqaciaacaGaaeqabaqabeGadaaakeaaiiGacuWF8oqBgaqcaaaa@2E79@ and σ^
 MathType@MTEF@5@5@+=feaafiart1ev1aaatCvAUfKttLearuWrP9MDH5MBPbIqV92AaeXatLxBI9gBaebbnrfifHhDYfgasaacH8akY=wiFfYdH8Gipec8Eeeu0xXdbba9frFj0=OqFfea0dXdd9vqai=hGuQ8kuc9pgc9s8qqaq=dirpe0xb9q8qiLsFr0=vr0=vr0dc8meaabaqaciaacaGaaeqabaqabeGadaaakeaaiiGacuWFdpWCgaqcaaaa@2E86@ are estimated mean and standard deviation, assuming that log intensity (log_10_*Cy3*) of individual features on an array followed normal distribution [30]. Thus, normalized *Cy3' *= *k · Cy3 *and *Cy5' = k · Cy5*. This intensity normalization does not change ratio of individual features. However, it permits establishment and validation of relationship between the absolute intensity *Cy3 *(arbitrary unit) of individual microarrays.

#### Lowess normalization

Normalizations using locally weighted linear regression and smooth scatter plot (Lowess) [31,32] were performed for each individual array to remove intensity-dependent dye-bias. An MA-plot is used to facilitate detecting intensity-dependent patterns in the log ratio, where *M *= log_2_(*Ratio*) and A=12log⁡10(Cy3⋅Cy5)
 MathType@MTEF@5@5@+=feaafiart1ev1aaatCvAUfKttLearuWrP9MDH5MBPbIqV92AaeXatLxBI9gBaebbnrfifHhDYfgasaacH8akY=wiFfYdH8Gipec8Eeeu0xXdbba9frFj0=OqFfea0dXdd9vqai=hGuQ8kuc9pgc9s8qqaq=dirpe0xb9q8qiLsFr0=vr0=vr0dc8meaabaqaciaacaGaaeqabaqabeGadaaakeaacqWGbbqqcqGH9aqpdaWcaaqaaiabigdaXaqaaiabikdaYaaacyGGSbaBcqGGVbWBcqGGNbWzdaWgaaWcbaGaeGymaeJaeGimaadabeaakiabcIcaOiabdoeadjabdMha5jabiodaZiabgwSixlabdoeadjabdMha5jabiwda1iabcMcaPaaa@41DD@. The Lowess smoother, implemented in the statistical software package R [33], locally normalizes *M *in an *A*-dependent manner log_2_(*Ratio'*) = log_2_(*Ratio*) *- L*(*A*), where *L*(*A*) is the Lowess fit to the MA-plot. The user defined parameters *f *(fraction of data used for smoothing at each point), *n*steps (number of iterations used in Lowess fit) are 0.5 and 3, respectively.

### Statistical analyses

We applied Significance Analysis of Microarrays (SAM, version 1.21) [21] to analyze the differential expression of genes by comparing expression profiles of *pog1Δ*, *ptp1-1*, or *ptp2-1 *with the wild type. At least 3 independently repeated microarray hybridizations were performed for reproducibility.

The completed microarray datasets are submitted to GEO databases (accession number GSE5941).

## Competing interests

The author(s) declares that there are no competing interests.

## Authors' contributions

ZC participated in the design of the study, performed the qRT-PCR and microarray analyses, and involved in the interpretation of the data. JTL, ME, KMK, KL participated in the statistical analysis of microarray data. JHL conceived the study, involved in design, analysis and interpretation, and drafted the manuscript.

All authors read and approved the final manuscript.

## Supplementary Material

Additional file 1 List of the up- and down-regulated genes. The table contained: (A) the list of up-regulated genes observed in all of the *pog1Δ*, *ptp1-1*, and *ptp2-1 *strains and (B) the list of down-regulated genes observed in any one of the *pog1Δ*, *ptp1-1*, and *ptp2-1 *strains. Click here for file

Additional file 2 List of RT-PCR primer sequences. The table contained nucleotide sequences of primers used in the RT-qPCR assays for comparing copy numbers of mtDNA with genomic DNA. Click here for file
